# Preparation of magnetic mesoporous silica nanoparticles as a multifunctional platform for potential drug delivery and hyperthermia

**DOI:** 10.1080/14686996.2016.1178055

**Published:** 2016-05-16

**Authors:** Xia Yu, Yufang Zhu

**Affiliations:** ^a^ School of Medical Instrument and Food Engineering, University of Shanghai for Science and Technology, Shanghai, P.R.China; ^b^ School of Materials Science and Engineering, University of Shanghai for Science and Technology, Shanghai, P.R.China

**Keywords:** Mesoporous nanoparticles, drug delivery, magnetic hyperthermia, multifunctionality, 30 Bio-inspired and biomedical materials, 102 Porous/Nanoporous/Nanostructured materials, 211 Scaffold/Tissue engineering/Drug delivery

## Abstract

We report the preparation of magnetic mesoporous silica (MMS) nanoparticles with the potential multifunctionality of drug delivery and magnetic hyperthermia. Carbon-encapsulated magnetic colloidal nanoparticles (MCN@C) were used to coat mesoporous silica shells for the formation of the core-shell structured MMS nanoparticles (MCN@C/mSiO_2_), and the rattle-type structured MMS nanoparticles (MCN/mSiO_2_) were obtained after the removal of the carbon layers from MCN@C/mSiO_2_ nanoparticles. The morphology, structure, magnetic hyperthermia ability, drug release behavior, *in vitro* cytotoxicity and cellular uptake of MMS nanoparticles were investigated. The results revealed that the MCN@C/mSiO_2_ and MCN/mSiO_2_ nanoparticles had spherical morphology and average particle sizes of 390 and 320 nm, respectively. The MCN@C/mSiO_2_ nanoparticles exhibited higher magnetic hyperthermia ability compared to the MCN/mSiO_2_ nanoparticles, but the MCN/mSiO_2_ nanoparticles had higher drug loading capacity. Both MCN@C/mSiO_2_ and MCN/mSiO_2_ nanoparticles had similar drug release behavior with pH-controlled release and temperature-accelerated release. Furthermore, the MCN@C/mSiO_2_ and MCN/mSiO_2_ nanoparticles showed low cytotoxicity and could be internalized into HeLa cells. Therefore, the MCN@C/mSiO_2_ and MCN/mSiO_2_ nanoparticles would be promising for the combination of drug delivery and magnetic hyperthermia treatment in cancer therapy.

## Graphical abstract

**Figure:**
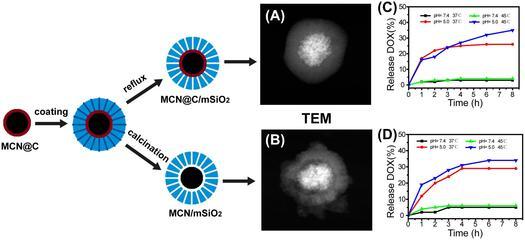

## 1. Introduction

Chemotherapy is often used for cancer therapy, but the side-effect of toxic free anticancer drugs is very serious to the body.[1] Therefore, the use of carriers for drug delivery is of great important and promising in cancer therapy.

To date, a variety of carriers including organic and inorganic materials have been studied for anticancer drug delivery.[2] Recently, mesoporous silica nanoparticles (MSNs) have been intensively studied as nanocarriers for drug delivery owing to their high surface area, tunable particle size and mesopore size, good biocompatibility and facial surface functionalization.[3–5] Compared to organic carriers such as liposomes, micelles and biodegradable polymers, MSNs can be free from various biochemical attacks. Furthermore, MSNs can load a high amount of drugs and control drug release behavior. For example, Niu et al. [6] developed a kind of core-shell MSN with smaller mesopore in the shell and larger mesopore in the core, which could achieve a three-step release behavior controlled by tuning the shell thickness from 5 nm to 60 nm^[6]^. Li et al. [7] designed PEGylated mesoporous silica nanorattles as nanocarriers for docetaxel delivery to liver cancer, and the docetaxel-loaded mesoporous silica nanorattles showed increased tumor inhibition ability compared to the clinical formulation of docetaxel^[7]^.

However, cancer recurrence often occurs when only chemotherapy is used to treat the tumors, although the use of carriers can allow targeted drug delivery and controlled drug release. Therefore, much effort has been made to enhance cancer therapeutic efficacy by combining other efficient approaches, such as gene therapy, immunotherapy, radiation therapy, and magnetic hyperthermia, to achieve a synergistic effect.[8–10] Among them, magnetic hyperthermia is considered an effective treatment option for cancer without adverse side effects. This method involves raising the temperature to 43–48°C to deactivate cancer cells,[11,12] where the temperature increase (or heat generation) is caused by magnetic nanoparticles (MNPs) due to the hysteresis loss and/or Néel and Brownian relaxations of MNPs under an alternating magnetic field.[10] Heat can increase the efficacy of different chemotherapeutic drugs in the hyperthermia temperature range.[13] On the other hand, studies demonstrated that MNPs-based hyperthermia treatment may induce anticancer immunity, and can also be used for controlled drug delivery.[14] The synergistic therapeutic effects may be achieved by the combination of chemotherapy with magnetic hyperthermia.[15–17] However, the critical issue is to prepare a multifunctional platform with the simultaneous controlled anticancer drug release and magnetic hyperthermia ability.

To solve this critical issue, magnetic mesoporous silica (MMS) nanoparticles are proposed to be a multifunctional platform for drug delivery and magnetic hyperthermia. Mesoporous silica can deliver drugs owing to the high drug loading in mesoporous channels.[3–5] MNPs can be embedded in mesoporous silica, which is useful for the enhancement of the stability of MNPs and the decrease of potential toxicity effect of MNPs on cells.[18] Therefore, preparation of MMS nanoparticles for potential drug delivery or magnetic hyperthermia has gained more attention.[19–30] Gai et al. [19] prepared fibrous-structured magnetic mesoporous particles (Fe_3_O_4_/FMSMs), which showed a sustained drug release behavior and excellent magnetic responsivity to external magnetic field. Furthermore, the Fe_3_O_4_/FMSMs carriers could deliver DOX into the cell cytoplasm and cell nucleus^[19]^. Shi and coworkers [20–22] reported a series of Fe_3_O_4_/Fe_2_O_3_@mSiO_2_ nanocapsules with a single or double mesoporous silica shell, and these nanocapsules had sufficient magnetic responsivity and high anticancer drug loading capacity^[20], [21], [22]^. Also, the DOX-loaded nanocapsules showed greater cytotoxicity to induce MCF-7 cell death than free DOX. Julian-Lopez et al. [23] for the first time reported mesoporous maghemite-organosilica microspheres with the multifunctionality of MR imaging and hyperthermia therapy^[23]^. Vallet-Regí and coworkers [24,25] synthesized magnetic γ-Fe_2_O_3_-encapsulated mesoporous silica microspheres, and these magnetic microspheres showed the controlled release of therapeutic drugs and good magnetic heating capacity under a low-frequency alternating magnetic field^[24], [25]^. Lu et al. [26] prepared magnetic iron oxide-loaded hollow mesoporous silica nanocapsules with a particle size of 100 nm, and they found that magnetic nanocapsules induced heating upon exposure to magnetic field and remotely triggered drug release^[26]^. However, there are limited studies on investigating MMS nanoparticles for drug delivery with synergistic magnetic hyperthermia until now.

Recently, we prepared Fe_3_O_4_/SiO_2_ MMS nanoparticles by encapsulating Fe_3_O_4_ nanoparticles in mesoporous silica using a sol-gel method, which had pH-controlled DOX release behavior and magnetic heating capacity under an alternating magnetic field.[27] Using DNA to cap the mesopore openings of MMS nanoparticles, the DOX-loaded nanosystem showed potential temperature-controlled DOX release induced by magnetic heating.[28] However, the Fe_3_O_4_ amount was limited in the prepared Fe_3_O_4_/SiO_2_ MMS nanoparticles, which results in the lower magnetic heating capacity.

In this study, we report a kind of MMS nanoparticle as a multifunctional platform for drug delivery with synergistic magnetic hyperthermia ability. Preparation of MMS nanoparticles involves the synthesis of carbon-encapsulated magnetic colloidal nanoparticles (MCN@C) by solvothermal treatment [31] and subsequently coating mesoporous silica on MCN@C to form the core-shell structured MCN@C/mSiO_2_ nanoparticles. Here, the carbon layer could enhance the ability to protect the magnetic colloidal nanoparticles from oxidation or dissolution in an acidic environment. After the treatment of the MCN@C/mSiO_2_ nanoparticles by calcination to remove the carbon layers, rattle-type MCN/mSiO_2_ nanoparticles were obtained. Furthermore, magnetic hyperthermia ability, drug release behavior, biocompatibility and cellular uptake of the MCN@C/mSiO_2_ and MCN/mSiO_2_ nanoparticles were investigated.

## 2. Experimental details

### 2.1. Materials

Ferrocene (Fe(C_5_H_5_)_2_, ≥98%), hydrogen peroxide (H_2_O_2_, 30%), acetone (C_3_H_6_O, ≥99%), cetyl trimethyl ammonium bromide (CTAB), triethanolamine (TEA) and tetraethyl orthosilicate (TEOS) were of analytic grade from the Sinopharm Chemical Reagent (Shanghai) Co., Ltd, Shanghai, China. Doxorubicin hydrochloride (DOX) was purchased from Sangon Biotech (Shanghai) Co., Ltd (Shanghai, China). Dodecane was obtained from TCI, Shanghai, China. All chemicals were used as received without further purification.

### 2.2. Preparation of carbon-encapsulated magnetic colloidal (MCN@C) nanoparticles

MCN@C nanoparticles were synthesized according to the previous report after minor modification.[31] Typically, 0.30 g of ferrocene was completely dissolved in 30 ml of acetone, and 1.50 ml of H_2_O_2_ was slowly added into the above solution to form the precursor solution. After the vigorous magnetic stirring for 30 min, the precursor solution was transferred to a Teflon-lined stainless autoclave, and then heated to 230°C for 48 h. After cooling to room temperature, the precipitates were collected by a magnet and washed with ethanol several times. Finally, the black products were dried at room temperature in a vacuum oven to obtain MCN@C nanoparticles.

### 2.3. Preparation of MMS nanoparticles

MMS nanoparticles were prepared via a one-pot biphase stratification approach after some modification.[32] Typically, 0.2 g of MCN@C nanoparticles was added into 60 ml of water, and followed the ultrasonication for 30 min to disperse MCN@C nanoparticles. Subsequently, the MCN@C suspension was transferred into a 100-ml round bottom flask including 0.5 g of CTAB and 0.36 g of TEA. After stirring gently at 60°C for 1 h, 20 ml of TEOS solution (5% v/v in dodecane) was carefully added to the flask. The reaction was kept at 60°C with continuous stirring for 8 h. Then, the colloidal nanoparticles were separated by centrifugation, and washed with ethanol several times. Finally, the obtained colloidal nanoparticles were treated in ammonium nitrate (NH_4_NO_3_) ethanol solution (0.6 wt%) at 60°C for 6 h thrice to remove the surfactant, which was denoted as MCN@C/mSiO_2_ nanoparticles. Rattle-type MMS nanoparticles were obtained by the calcination of the MCN@C/mSiO_2_ nanoparticles at 540°C for 7 h, which was denoted as MCN/mSiO_2_ nanoparticles.

### 2.4. Characterization

The wide-angle X-ray diffraction (WAXRD) patterns were performed on a D8 ADVANCE powder diffractometer (Bruker, Germany) using Cu Kα1 radiation (1.5405 Å). Scanning electron microscope (SEM) observations were carried out with a FEI Quanta 450 field emission setup. Scanning transmission electron microscopy (STEM) images were obtained with a Tecnai G2 F30 electron microscope (FEI, Netherlands) operated at an acceleration voltage of 300 kV. UV-vis analysis was performed on a NanoDrop2000C spectrophotometer (Thermo Fisher Scientific, USA). N_2_ sorption measurements were carried out on a Micromeritics Tristar 3020 automated surface area and pore size analyzer (Micromeritics, USA) at 77 K under continuous adsorption conditions. Brunauer–Emmett–Teller (BET) and Barrett–Joyner–Halenda (BJH) methods were used to determine the surface area and pore size distribution. Magnetization curves were carried out using a LakeShore 7407 vibrating sample magnetometer (VSM, Lake Shore, USA) at 298 K.

### 2.5. Drug loading and release from MMS nanoparticles

In this study, anticancer drug (doxorubicin hydrochloride, DOX) was introduced into the MCN@C/mSiO_2_ and MCN/mSiO_2_ nanoparticles to verify drug release behavior. For drug loading, DOX was dissolved into PBS to obtain DOX solution with a concentration of 0.5 mg ml^–1^. Subsequently, 60 mg of MCN@C/mSiO_2_ or MCN/mSiO_2_ nanoparticles was dispersed into 12 ml of DOX solution. The mixture solution was agitated for 24 h under dark conditions, and then collected by centrifugation to obtain the DOX-loaded MCN@C/mSiO_2_ or MCN/mSiO_2_ nanoparticles. The DOX-loaded nanoparticles were washed with PBS twice to remove DOX adsorbed on the surfaces. The DOX loading amount was determined by measuring the initial and residual DOX solutions using UV-vis analysis at the wavelength of 481 nm.

The DOX release behaviors from the MCN@C/mSiO_2_ or MCN/mSiO_2_ nanoparticles were performed as follows: 60 mg of the DOX-loaded MCN@C/mSiO_2_ or MCN/mSiO_2_ nanoparticles was dispersed in 10 ml of the release medium (pH 5.0 or pH 7.4 at 37°C and pH 5.0 or pH 7.4 at 45°C). At the predetermined time intervals, 20 μl of the released solution was withdrew and centrifuged for UV-vis analysis. After the UV-vis analysis, the residual solution was returned to the release system.

### 2.6. Magnetic hyperthermia ability of MMS nanoparticles

The evaluation of magnetic hyperthermia ability of the MCN@C, MCN@C/mSiO_2_ and MCN/mSiO_2_ nanoparticles was carried out on a DM100 System (NanoScale Biomagnetics, Spain). The MCN@C, MCN@C/mSiO_2_ and MCN/mSiO_2_ nanoparticles were dispersed in H_2_O, and the concentration was 30 mg ml^–1^, respectively. Subsequently, 1.0 ml of the nanoparticles suspension was put in the test vessel and then fixed under an alternating magnetic field. The magnetic field strength and frequency were set to be 180 Gauss and 409 kHz, respectively. The specific absorption rate (SAR) was calculated to evaluate the magnetic hyperthermia ability of the MCN@C, MCN@C/mSiO_2_ and MCN/mSiO_2_ nanoparticles.

### 2.7. Cell culture

In this study, HeLa cells lines were used to test *in vitro* cytotoxicity and cellular uptake of MMS nanoparticles. HeLa cell lines were maintained in MEM medium containing 10% fetal bovine serum, 100 units ml^–1^ penicillin, and 100 mg ml^–1^ streptomycin. Cells were cultured with the complete medium in 5% CO_2_ at 37°C.

### 2.8. *In vitro* cytotoxicity of MMS nanoparticles

*In vitro* cytotoxicities of the MCN@C, MCN@C/mSiO_2_ and MCN/mSiO_2_ nanoparticles were evaluated using Cell Counting Kit-8 (CCK-8) assay (Dojindo, Japan). Before cell seeding, the MCN@C, MCN@C/mSiO_2_ and MCN/mSiO_2_ nanoparticles were dispersed in MEM medium with a centration of 1 mg ml^–1^. HeLa cells were seeded into a 96-well plate at a density of 5000 cells in each well. Subsequently, the nanoparticles suspension were added in each well, and the final concentrations of the MCN@C, MCN@C/mSiO_2_ and MCN/mSiO_2_ nanoparticles were 0, 25, 50, 75 and 100 μg ml^–1^ with the medium volume of 100 μl in each well. After 24 h incubation of cells, 10 μl of CCK-8 solution was added into each well, and the cells were incubated for another 3 h. The optical density (OD) was then measured by the absorbance at 450 nm using a microplate reader (Bio-Rad 680, California, USA).

### 2.9. Cellular uptake of MMS nanoparticles

For observation of cellular uptake of the MCN@C, MCN@C/mSiO_2_ and MCN/mSiO_2_ nanoparticles, three kinds of nanoparticles were labeled with rhodamine B isothiocyanate (RBITC) to form RBITC-labeled nanoparticles. Typically, 1.0 × 10^5^ cells were seeded in a 35-mm Petri dish. After the cells were attached to the bottom of Petri dish they were washed twice with PBS. Subsequently, RBITC-labeled nanoparticles were added to the Petri dishes (100 μg ml^–1^). After another 4 h incubation, the cells were washed with PBS three times to remove the remaining nanoparticles and dead cells. Then, the nuclei of cells were stained for 25 min using Hoechst 33342 solution (0.5 μg ml^–1^). After washing with PBS to remove the residual dye molecules, the cells were fixed with 4% of paraformaldehyde for 20 min. Finally, the cells were washed with PBS for three times, and observed under a confocal laser scanning microscopy (SP5, Leica, Hamburg, Germany).

## 3. Results and discussion

### 3.1. Characterization of MCN@C, MCN@C/mSiO_2_ and MCN/mSiO_2_ nanoparticles

**Figure 1.**
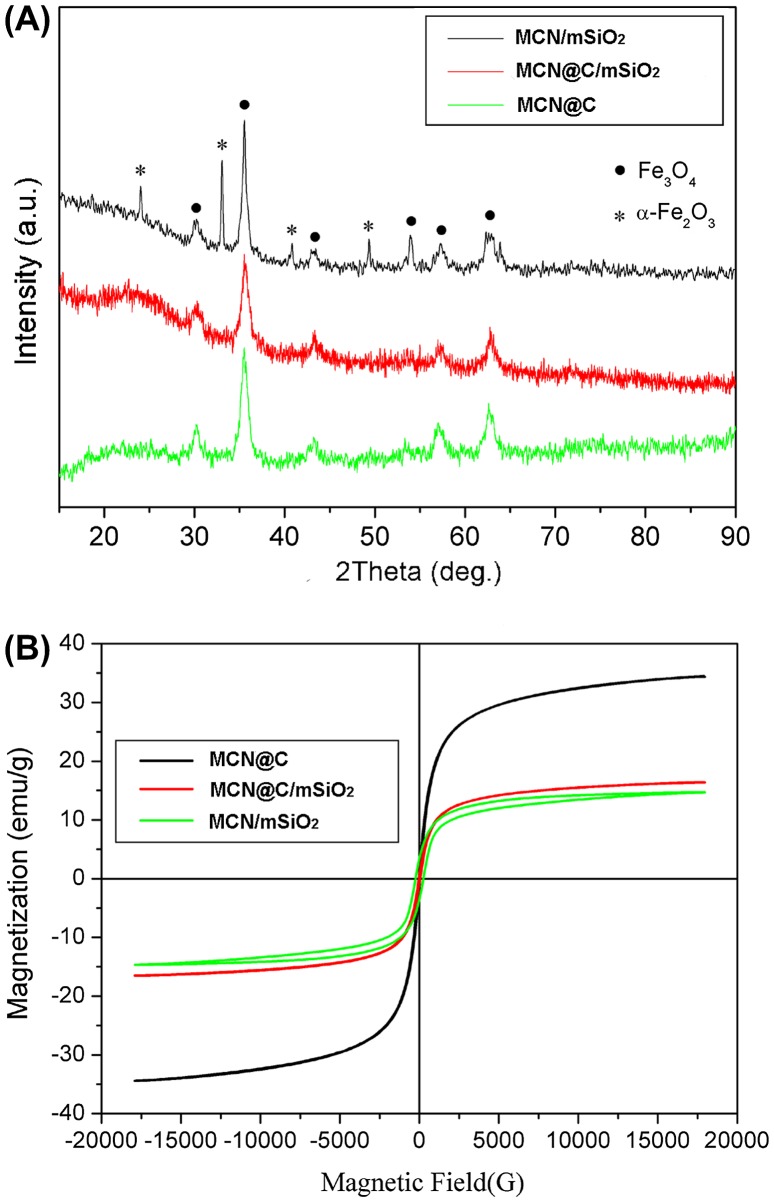
The wide-angle XRD patterns (A) and magnetization curves measured at room temperature (B) of the MCN@C, MCN@C/mSiO_2_ and MCN/mSiO_2_ nanoparticles.

The wide-angle XRD patterns of MCN@C, MCN@C/mSiO_2_ and MCN/mSiO_2_ nanoparticles are shown in Figure 1(A). MCN@C nanoparticles were synthesized by the previously reported method,[31] and the XRD pattern indicated that the MCN@C nanoparticles mainly had magnetite (JCPDS file 19-0629) or maghemite (JCPDS file 39-1346). For MCN@C/mSiO_2_ nanoparticles, the XRD pattern showed a broad reflection at 2*θ* = 20–25°, suggesting an amorphous structure of the mesoporous silica coating; but other diffraction peaks were the same as those of MCN@C nanoparticles, indicating that the crystalline structure of the MCN@C nanoparticles did not change after the coating of mesoporous silica and the removal of surfactant. But for MCN/mSiO_2_ nanoparticles, several diffraction peaks indexed to hematite (JCPDS file 33-664) were observed on the XRD pattern, except for the peaks for magnetite or maghemite, which suggests that some magnetite or maghemite in MCN@C nanoparticles have converted to hematite in MCN/mSiO_2_ nanoparticles due to the calcination treatment for the removal of carbon and surfactant. On the other hand, compared to the MCN@C and MCN@C/mSiO_2_ nanoparticles, the width of the diffraction peaks of the MCN/mSiO_2_ nanoparticles became much narrower, which suggested that the calcination treatment of the MCN@C/mSiO_2_ nanoparticles induced the formation of larger crystals of magnetite or maghemite. According to Scherrer’s formula, the magnetic nanocrystal size was increased from 16.1 nm in the MCN@C/mSiO_2_ nanoparticles to 18.2 nm in the MCN/mSiO_2_ nanoparticles.

Figure 1(B) shows the magnetization curves of the MCN@C, MCN@C/mSiO_2_ and MCN/mSiO_2_ nanoparticles measured at 298 K with a vibrating sample magnetometer (VSM). Similar to the previous reports,[31] the magnetic property of the MCN@C nanoparticles demonstrated a very small remanence and reveals its superparamagnetic nature at 298 K, and the magnetization saturation value (*M*_*s*_) of the MCN@C nanoparticles was estimated to be 34.4 emu g^–1^. It can also be observed that a very small hysteresis loop is for the MCN@C/mSiO_2_ nanoparticles and the *M*_*s*_ decreased to be 16.5 emu g^–1^. This indicated that the MCN@C nanoparticles still maintained superparamagnetic behavior. The decrease in *M*_*s*_ was attributed to the non-magnetic mesoporous silica coating on the MCN@C nanoparticles. However, a relatively obvious hysteresis loop was observed in the magnetization curve of the MCN/mSiO_2_ nanoparticles. It might be that the larger crystals of magnetite or maghemite and hematite formation contributed the ferromagnetic behavior of the MCN/mSiO_2_ nanoparticles. On the other hand, the *M*_*s*_ of the MCN/mSiO_2_ nanoparticles decreased to be 14.7 emu g^–1^ owing to the formation of some hematite crystals from magnetite or maghemite during the calcination treatment. Compared to our previously reported MMS nanoparticles (*M*_*s*_ of 4.2–8.1 emu g^–1^),[27] the MCN@C/mSiO_2_ and MCN/mSiO_2_ nanoparticles exhibited much higher magnetization saturation, which might enhance the magnetic heating capacity.

**Figure 2.**
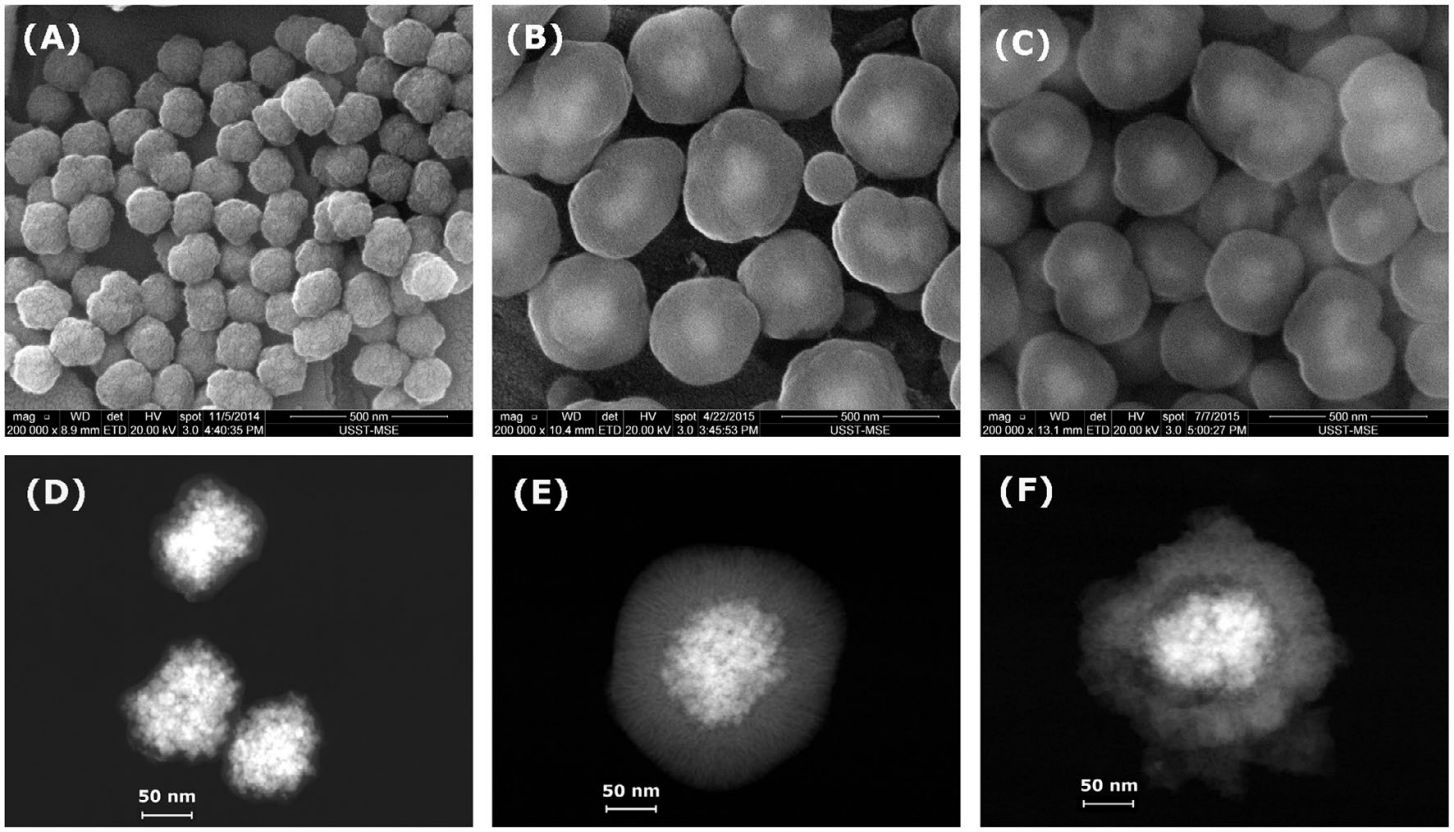
SEM and STEM images of the MCN@C (A, D); MCN@C/mSiO_2_ (B, E); and MCN/mSiO_2_ (C, F) nanoparticles.

Figure 2 shows SEM and STEM images of MCN@C, MCN@C/mSiO_2_ and MCN/mSiO_2_ nanoparticles. The MCN@C nanoparticles had a narrow particle size distribution, and the average particle size was about 170 nm (Figure 2(A)). STEM images revealed that the MCN@C nanoparticles had an obvious core-shell structure, similar to the previous reports,[31] and magnetic cores were composed of small magnetite or maghemite nanocrystals and thin carbon shells were coated on the surfaces of magnetic cores. After coating with mesoporous silica on the MCN@C nanoparticles, the MCN@C/mSiO_2_ and MCN/mSiO_2_ nanoparticles still had spherical morphology, but the average particle size increased to about 390 and 320 nm, respectively. STEM images showed different structures for the MCN@C/mSiO_2_ and MCN/mSiO_2_ nanoparticles. The MCN@C/mSiO_2_ nanoparticles had the core/shell structure, while the MCN/mSiO_2_ nanoparticles had a rattle-type structure, e.g. iron oxide nanocrystals were encapsulated in hollow cores of the MCN/mSiO_2_ nanoparticles. On the other hand, dendritic mesoporous channels can be clearly observed on both MCN@C/mSiO_2_ and MCN/mSiO_2_ nanoparticles, indicating the possibility of drug loading in the MCN@C/mSiO_2_ and MCN/mSiO_2_ nanoparticles for drug delivery.

**Figure 3.**
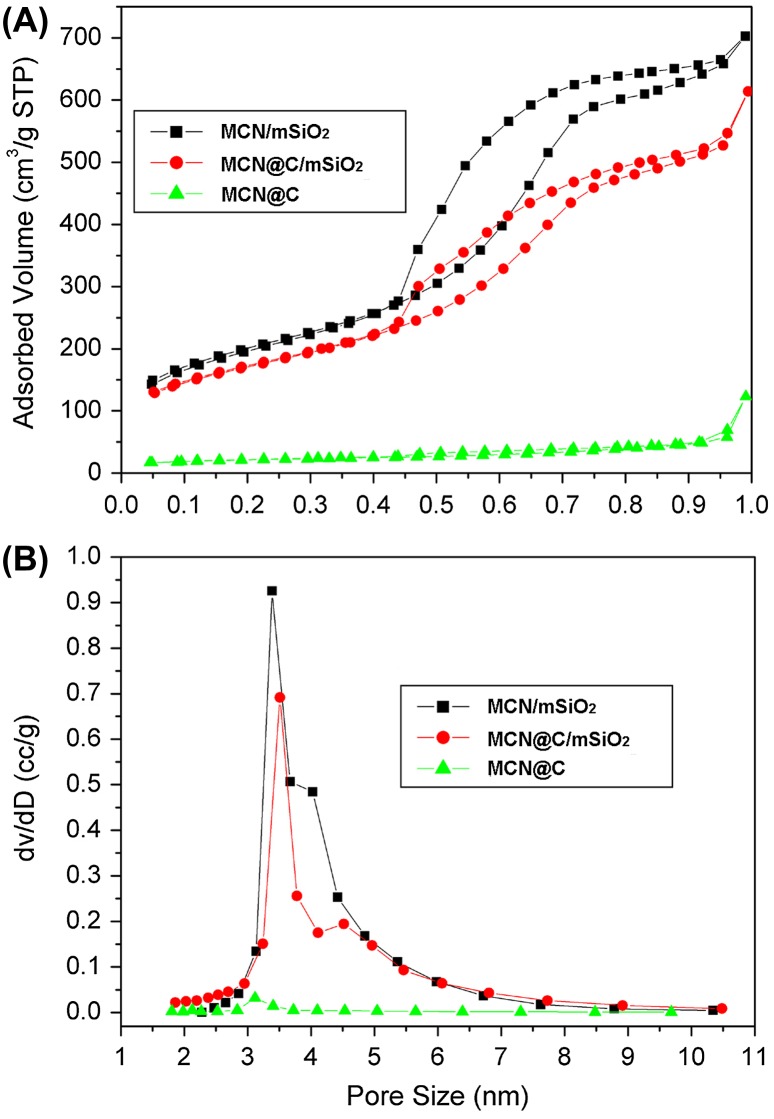
N_2_ adsorption-desorption isotherms (A) of the MCN@C, MCN@C/mSiO_2_ and MCN/mSiO_2_ nanoparticles and their corresponding pore size distribution curves (B).

Figure 3 shows N_2_ adsorption–desorption isotherms of the MCN@C, MCN@C/mSiO_2_ and MCN/mSiO_2_ nanoparticles and their corresponding pore size distribution curves. The MCN@C nanoparticles had much lower N_2_ adsorption and no mesopore size distribution, the BET surface area (S_BET_) was about 70 m^2^ g^–1^. However, both isotherms of the MCN@C/mSiO_2_ and MCN/mSiO_2_ nanoparticles belong to type IV isotherm, indicating the mesoporous structure of the MCN@C/mSiO_2_ and MCN/mSiO_2_ nanoparticles. The S_BET_ of the MCN@C/mSiO_2_ and MCN/mSiO_2_ nanoparticles were 606 and 709 m^2^ g^–1^, respectively. The pore volumes (V_P_) at P/P_0_ = 0.97 for the MCN@C/mSiO_2_ and MCN/mSiO_2_ nanoparticles were 0.95 and 1.08 cm^3^ g^–1^, respectively (Table 1). However, the MCN@C/mSiO_2_ and MCN/mSiO_2_ nanoparticles exhibited the similar pore size distributions peaked at 3.4 and 3.2 nm. Therefore, the coating of mesoporous silica on the MCN@C nanoparticles provides high surface area and mesoporous channels for loading a high amount of drugs. Furthermore, the MCN/mSiO_2_ nanoparticles created hollow cores and might contribute much higher drug loading capacity.

**Table 1.** The structure parameters, DOX loading capacities and SAR values of the MCN@C, MCN@C/mSiO_2_ and MCN/mSiO_2_ nanoparticles.

Samples	S_BET_ (m^2^ g^–1^)	V_p_ (cm^3^ g^–1^)	D_p_ (nm)	DOX loading (μg/mg)	SAR (W g^–1^)
MCN@C	70	0.16	/	/	54.7
MCN@C/mSiO_2_	606	0.95	3.4	58.5	34.8
MCN/mSiO_2_	709	1.08	3.5	96.2	25.7

### 3.2. Magnetic hyperthermia ability of the MCN@C, MCN@C/mSiO_2_ and MCN/mSiO_2_ nanoparticles

**Figure 4.**
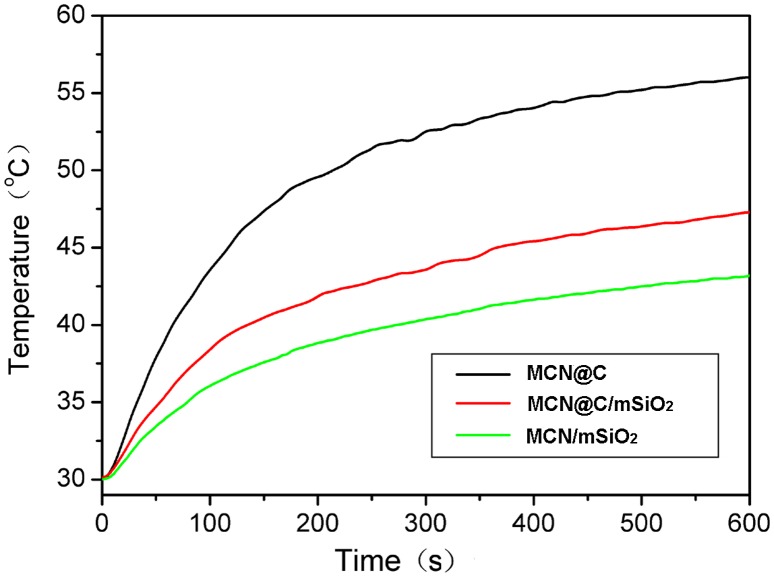
The magnetic heating curves of the MCN@C, MCN@C/mSiO_2_ and MCN/mSiO_2_ nanoparticles with a concentration of 30 mg ml^–1^ in H_2_O evaluated under an alternating magnetic field. The magnetic field strength and frequency are 180 Gauss and 409 kHz, respectively.

Figure 4 shows the magnetic heating curves of the MCN@C, MCN@C/mSiO_2_ and MCN/mSiO_2_ nanoparticles with a concentration of 30 mg ml^–1^ evaluated under the same alternating magnetic field. From the temperature kinetic curve of the MCN@C nanoparticles, the temperature increased from 37 to 59°C within 5 min, suggesting that alternating magnetic field can induce the MCN@C nanoparticles to generate heat and reach the hyperthermia temperature within a very short time. For the MCN@C/mSiO_2_ and MCN/mSiO_2_ nanoparticles, the temperature increase with time was a little slower than the MCN@C nanoparticles. But the absolute temperature increases in 5 min for the MCN@C/mSiO_2_ and MCN/mSiO_2_ nanoparticles were estimated to be 10.4 and 13.5°C, respectively. That is to say, the MCN@C/mSiO_2_ and MCN/mSiO_2_ nanoparticles can also generate heat for hyperthermia treatment. Correspondingly, the specific absorption rate (SAR) of the MCN@C, MCN@C/mSiO_2_ and MCN/mSiO_2_ nanoparticles, which was quantified for the magnetic heating capacity of magnetic materials, was estimated to be 54.7, 34.8 and 25.7 W g^–1^, respectively. The MCN@C/mSiO_2_ and MCN/mSiO_2_ nanoparticles also showed good magnetic hyperthermia ability, although the magnetic hyperthermia ability of the MCN@C/mSiO_2_ and MCN/mSiO_2_ nanoparticles was lower than that of the MCN@C nanoparticles, owing to the coating of non-magnetic mesoporous silica on the MCN@C nanoparticles. On the other hand, the MCN@C/mSiO_2_ nanoparticles showed a little higher magnetic heating capacity than the MCN/mSiO_2_ nanoparticles, which is attributed to the maintaining of iron oxide crystals in the MCN@C/mSiO_2_ nanoparticles during extracting treatment, but a small amount of hematite crystals were formed in the MCN/mSiO_2_ nanoparticles during the calcination treatment.

### 3.3. Drug release behavior from the MCN@C/mSiO_2_ and MCN/mSiO_2_ nanoparticles

Doxorubicin hydrochloride (DOX) was used as a model anticancer drug to investigate the drug release behavior from the MCN@C/mSiO_2_ and MCN/mSiO_2_ nanoparticles in this study. The DOX loading amounts in the MCN@C/mSiO_2_ and MCN/mSiO_2_ nanoparticles were 58.5 and 96.2 μg DOX per 1 mg of the MCN@C/mSiO_2_ or MCN/mSiO_2_ nanoparticles, respectively. Here, the higher DOX loading capacity of the MCN/mSiO_2_ nanoparticles was attributed to its higher surface area and hollow cavity.

**Figure 5.**
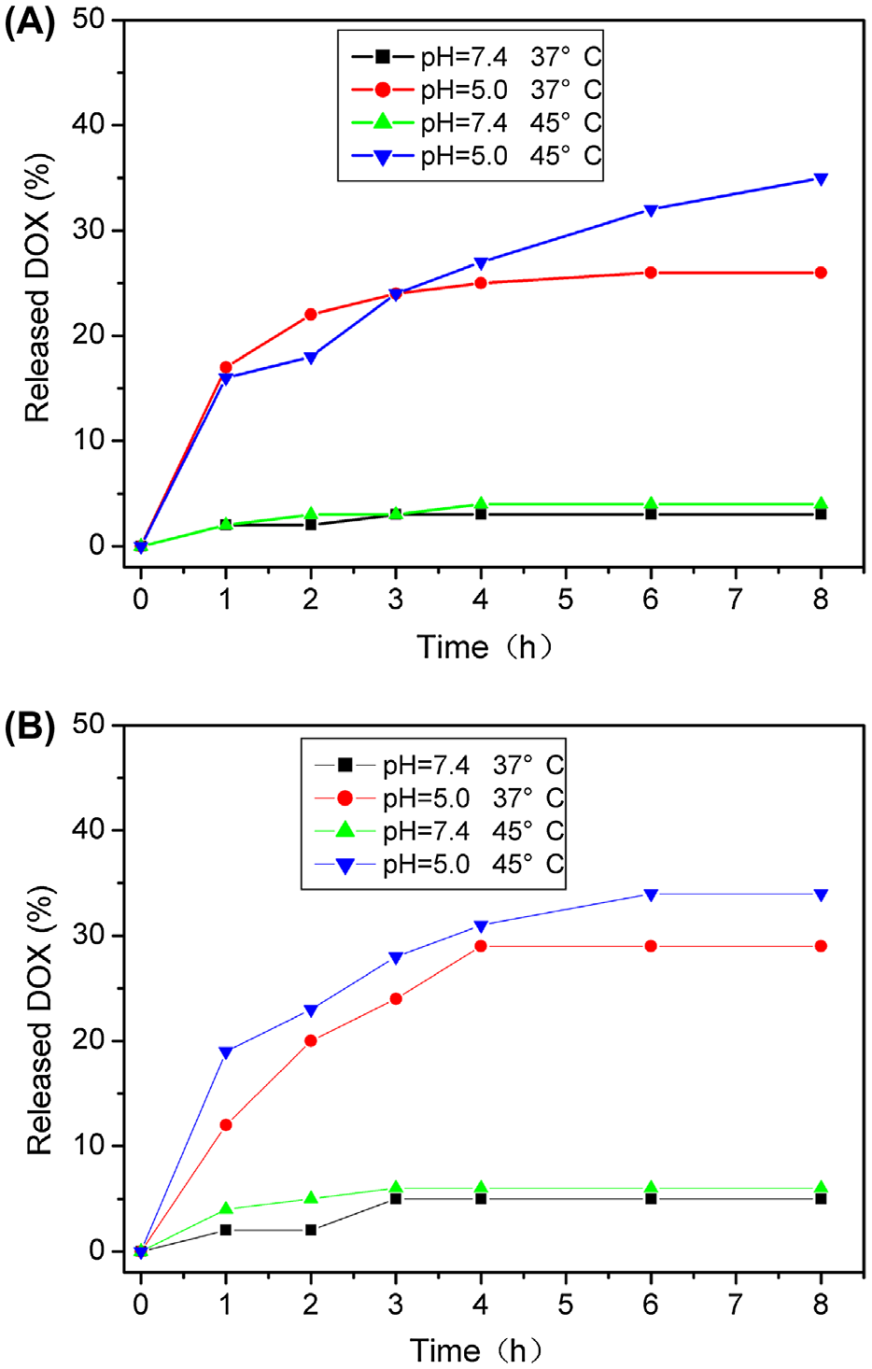
The DOX release behaviors from the MCN@C/mSiO_2_ (A) and MCN/mSiO_2_ (B) nanoparticles in the release media of pH 7.4 and 5.0 and at temperatures of 37 and 45°C.

Figure 5 shows the DOX release profiles from the MCN@C/mSiO_2_ and MCN/mSiO_2_ nanoparticles in the release media with pH 7.4 and 5.0 at temperatures of 37 and 45°C. Here, the temperature of 45°C was used to evaluate the possibility of drug release in the range of hyperthermia temperature. Both MCN@C/mSiO_2_ and MCN/mSiO_2_ nanoparticles exhibited pH dependent DOX release behavior. The DOX releases from the MCN@C/mSiO_2_ and MCN/mSiO_2_ nanoparticles were very slow in pH 7.4 medium (only *c*.2–5% of DOX release in 8 h), but the DOX release became faster in pH 5.0 medium (*c*.25–30% of DOX release in 8 h). The DOX release behaviors from the MCN@C/mSiO_2_ and MCN/mSiO_2_ nanoparticles are similar to those in previous reports on the DOX release from mesoporous silica nanoparticles.[27] It has been accepted that the electrostatic interaction and hydrogen bonding between DOX molecules and the surfaces of silica nanoparticles could become weaker with lowering pH value, owing to the protonation of surface silanols. Thus, DOX molecules are easy to detach from the surfaces of silica nanoparticles in acidic environment, and thereby fasten DOX release.

On the other hand, for both MCN@C/mSiO_2_ and MCN/mSiO_2_ nanoparticles, the DOX release at 45°C was slightly faster compared to that at 37°C, which might be because the increased temperature can accelerate the diffusion of DOX molecules in the mesoporous channels. In this study, the MCN@C/mSiO_2_ and MCN/mSiO_2_ nanoparticles can generate heat under an alternating magnetic field to raise temperature, and thereby stimulate drug diffusion.

Therefore, such drug release behavior could decrease the side-effect of toxic anticancer drug delivery, because anticancer drugs mostly do not release from the carriers during the circulation of the drug delivery system in the blood stream (pH 7.4). When the drug delivery system is internalized in the targeted cancer cells, faster DOX release could enhance the therapeutic efficiency owing to the acidic environment in the endosome/lysosome and cytosol of cancer cells (pH 5.0–5.5). The combination with magnetic hyperthermia could further accelerate drug release after the endocytosis of drug delivery system by cancer cells.

### 3.4. *In vitro* cytotoxicity and cellular uptake of MMS nanoparticles

**Figure 6.**
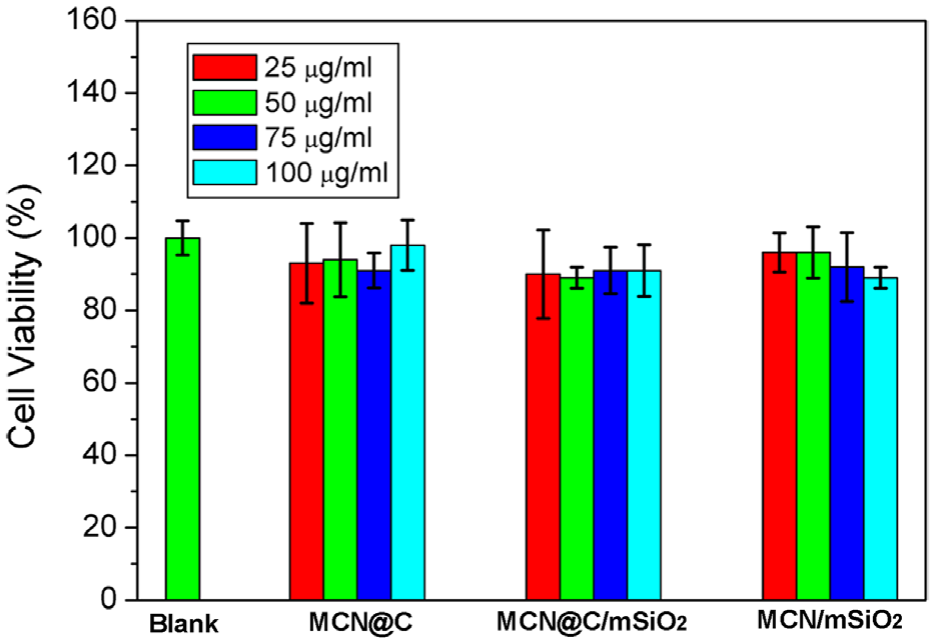
Viabilities of the MCN@C, MCN@C/mSiO_2_ and MCN/mSiO_2_ nanoparticles to HeLa cells after incubation for 24 h evaluated using CCK-8 assay.

Figure 6 shows *in vitro* cytotoxicities of the MCN@C, MCN@C/mSiO_2_ and MCN/mSiO_2_ nanoparticles against HeLa cells evaluated using CCK-8 assay. Cell viabilities of the MCN@C, MCN@C/mSiO_2_ and MCN/mSiO_2_ nanoparticles were more than 90% even up to a concentration of 100 μg ml^–1^, and they had no significant difference compared to those of the control group. The results revealed that the MCN@C, MCN@C/mSiO_2_ and MCN/mSiO_2_ nanoparticles are safe for drug delivery.

**Figure 7.**
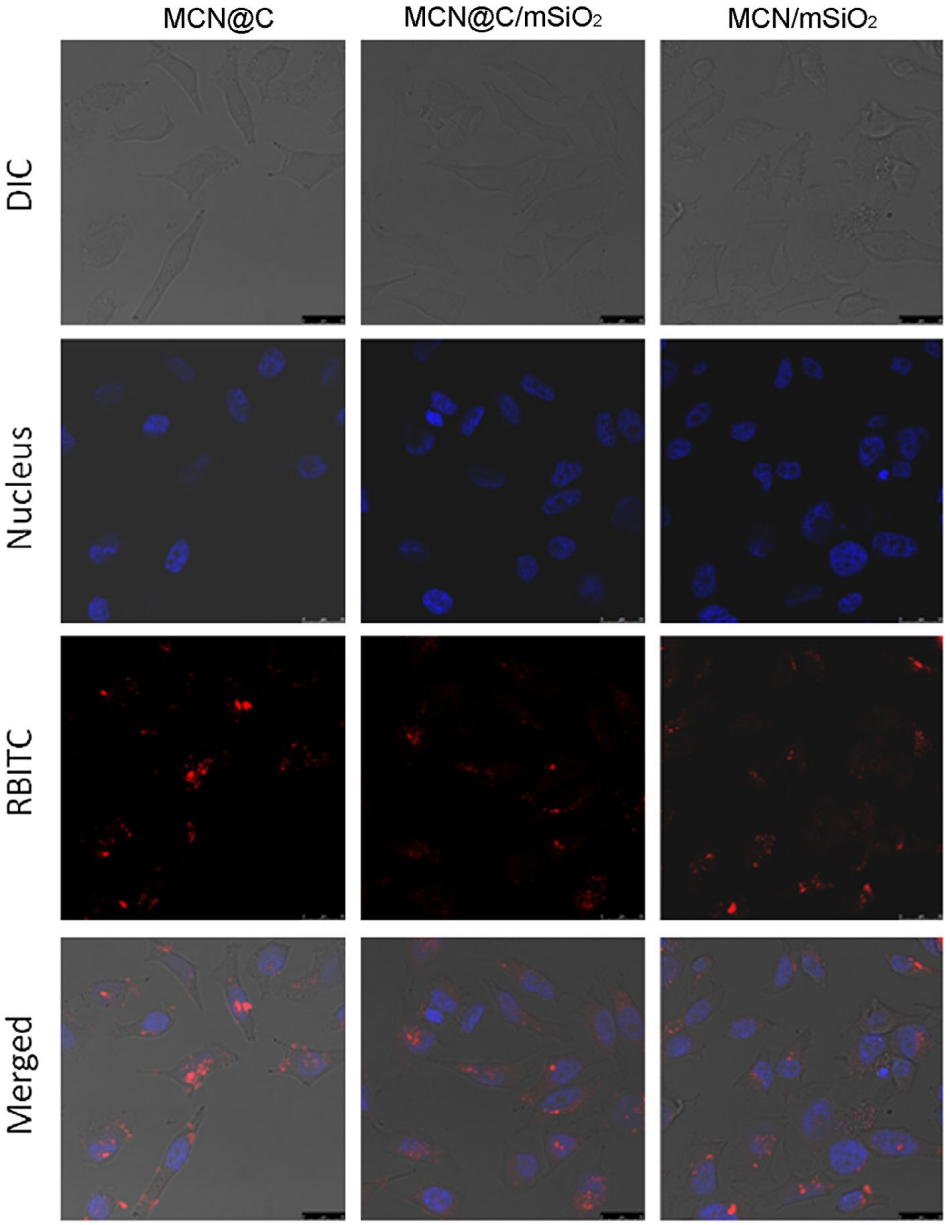
Cellular uptake of the MCN@C, MCN@C/mSiO_2_ and MCN/mSiO_2_ nanoparticles in HeLa cells after 4 h of incubation with a concentration of 100 μg ml^–1^: DIC shows the optical photograph of HeLa cells; Nucleus shows the nuclei of HeLa cells stained with Hoechst 33342; RBITC shows the location of RBITC-labeled nanoparticles; Merged shows the overlap DIC, Nucleus and RBITC images, indicating that RBITC-labeled MCN@C, MCN@C/mSiO_2_ and MCN/mSiO_2_ nanoparticles were taken up by HeLa cells. All scale bars are 25 μm.

Cellular uptake of the drug carriers is very important for enhancing the drug delivery efficiency. On the one hand, cellular uptake of drug carriers could be beneficial for magnetic hyperthermia therapy owing to magnetic local heating in cancer cells. On the other hand, cellular uptake of drug carriers could also enhance the chemotherapeutic efficiency due to the intracellular delivery of drugs. In this study, RBITC-labeled MCN@C, MCN@C/mSiO_2_ and MCN/mSiO_2_ nanoparticles were incubated with HeLa cells for 4 h to observe the cellular uptake. As shown in Figure 7, red fluorescence could be observed in the cells for each type of magnetic mesoporous nanoparticles, which suggests that the MCN@C, MCN@C/mSiO_2_ and MCN/mSiO_2_ nanoparticles were taken up into HeLa cells after endocytosis. However, the MCN@C, MCN@C/mSiO_2_ and MCN/mSiO_2_ nanoparticles have not been internalized into the nuclei. Furthermore, the distributions of red fluorescence had no significant difference among the MCN@C, MCN@C/mSiO_2_ and MCN/mSiO_2_ nanoparticles. Therefore, the MCN@C/mSiO_2_ and MCN/mSiO_2_ nanoparticles as nanocarriers for anticancer drug delivery could achieve local drug delivery and magnetic hyperthermia, and thereby enhance the cancer therapeutic efficiency.

## 4. Conclusions

In this study, we have synthesized MMS nanoparticles (MCN@C/mSiO_2_ and MCN/mSiO_2_) as a multifunctional platform for potential drug delivery and magnetic hyperthermia. The MCN@C/mSiO_2_ nanoparticles had core-shell structure with higher magnetic heating capacity compared to the MCN/mSiO_2_ nanoparticles, but the MCN/mSiO_2_ nanoparticles had higher drug loading capacity. Both MCN@C/mSiO_2_ and MCN/mSiO_2_ nanoparticles had similar drug release behavior with pH-controlled release and temperature-accelerated release. Furthermore, the MCN@C/mSiO_2_ and MCN/mSiO_2_ nanoparticles showed low cytotoxicity and could be internalized into cells within a short period. Therefore, the MCN@C/mSiO_2_ and MCN/mSiO_2_ nanoparticles would be promising for chemotherapy with synergistic magnetic hyperthermia in cancer therapy.

## Disclosure statement

No potential conflict of interest was reported by the authors.
